# The Impact of the COVID-19 Pandemic on Sense of Belonging and Science Outcomes among Biomedical Science Students: A Longitudinal Study

**DOI:** 10.3390/educsci13060579

**Published:** 2023-06-05

**Authors:** Patricia Escobedo, Sungmin Moon, Kyle Moreno, Judith C. P. Lin, Patchareeya P. Kwan, Gilberto E. Flores, Gabriela Chavira

**Affiliations:** 1 Department of Psychology, California State University Northridge, Northridge, CA 91330, USA; 2 Health Equity Research and Education Center, California State University Northridge, Northridge, CA 91330, USA; 3 Department of Health Sciences, California State University Northridge, Northridge, CA 91330, USA; 4 Department of Biology, California State University Northridge, Northridge, CA 91330, USA

**Keywords:** undergraduate research experiences (URE), undergraduates, STEM, biomedical science, COVID-19, science identity, science self-efficacy, sense of belonging, Hispanic-serving institution (HSI)

## Abstract

To understand how COVID-19 impacted undergraduate research experiences (URE), the current study examined how student outcomes changed over time among biomedical science majors. In addition, this study describes how a Building Infrastructure Leading to Diversity (BUILD) Promoting Opportunities for Diversity in Education and Research (PODER) URE program shifted entirely online in response to COVID-19. Biomedical science majors at a university in Southern California completed surveys in 2019 and 2020 and rated their science identity, science self-efficacy, and academic self-concept. We examined how scores changed over time by comparing: (1) BUILD and non-BUILD students and (2) students from underrepresented groups (URG) and non-URG students. Sense of belonging scores from 2020 were also compared among BUILD and non-BUILD students. BUILD students reported a significant increase in science self-efficacy scores, unlike non-BUILD participants. BUILD students also increased their science identity scores, unlike non-BUILD participants. Differences in sense of belonging were not significant, and differences between URG and non-URG students were not significant. Given the importance of science self-efficacy and science identity in a student’s academic trajectory, our results indicate that UREs such as BUILD PODER were able to improve or maintain critical student outcomes during a pandemic. These results highlight the importance of URE participation among biomedical science majors.

## 1. Introduction

It is well established that women and members from underrepresented groups (URG), such as Black, American Indian/Alaska Native, Latinx, and Pacific Islanders, are underrepresented in biomedical and science, technology, engineering, and math (STEM) fields in the United States [1,2]. According to the National Center for Science and Engineering Statistics (NCSES) [2], approximately 34.9 million people work in STEM occupations. From 2011 to 2021, women in STEM increased from 32% to 35%, while URGs in STEM increased from 18.4% to 24.6% [2]. Despite these gains, the STEM workforce remains predominantly male (65%) and White (64%) [2]. While the proportion of women (35%), Hispanic/Latinx (15%), Black (9%), and Native American/Alaska Native (0.6%) in STEM occupations are at the highest levels seen in the past decade, underrepresentation by gender and race/ethnicity remains a challenge [2].

Efforts to improve recruitment and retention of women and URGs include education and training programs designed to challenge the stereotype of who can be a STEM professional [3,4] or in STEM training [1]. In addition, these programs may improve a student’s sense of belonging in STEM and increase URGs’ persistence in STEM [4,5]. Over the past 30 years, increased efforts by U.S. federal agencies to diversify STEM have yielded successful equity initiatives and undergraduate research experiences (UREs) for college students from URGs [6,7].

### 1.1. Undergraduate Research Experiences

UREs allow students to engage in scientific research and prepare for a career in biomedical and STEM fields. Many traditional UREs pair undergraduates with faculty mentors [8–10], are designed to build a sense of community among URE participants [8], and provide graduate school and career preparation [10,11]. UREs may also offer academic curricula to students that would otherwise be hidden. The hidden curriculum refers to “the structure and culture of schooling [that] systemically advantage privileged students over their peers from systematically marginalized groups” [12] (p. 1). Through URE participation, students can address these inequities by working with faculty and advanced peers who help them connect with institutional resources (e.g., courses, programs, scholarships) and networking opportunities [9].

Undergraduate research experiences are an important strategy to retain college students in STEM-related majors. For example, UREs have been found to increase student persistence in STEM [13,14] and increase commitment to pursue scientific careers [7,15–17]. A longitudinal sample of 3300 undergraduate STEM students, which included social and behavioral sciences, compared students who participated in a funded URE (i.e., NSF, NIH, NASA) to non-URE peers and found that URE participation increased student understanding of research design and implementation, confidence in research skills, and awareness of graduate school expectations [18]. In addition, URE participation clarified students’ interests in STEM careers and increased interest in earning a Ph.D. in the future [18].

### 1.2. Science Identity

Developing and maintaining a strong science identity can improve a student’s likelihood to persist in STEM disciplines [19–21]. Carlone and Johnson [19] identified three important components of science identity: (1) recognition—recognition by self and meaningful others as a “science person”, (2) performance—the utilization of scientific practices and tools, and (3) competence—the knowledge and understanding of science concepts. Mentors, play a significant role in helping students feel like scientists [22,23]. Being recognized and validated by faculty, mentors, and peers encourage a student’s self-recognition as a scientist [19].

Several studies found that UREs that provide students with direct mentorship increased levels of science identity among participating students, especially students from URGs [3,10,15,16,24]. Camacho et al. [15] examined differences in science identity among URE and non-URE Latinx students, and findings indicated that students participating in the URE with a mentor reported the highest levels of science identity. Additionally, previous research found that STEM majors who entered college with lower science identities and experienced declines in science identity during college were less likely to obtain a science-related career after graduation [25]. These findings indicate that science identity is an important predictor of pursuing STEM careers.

### 1.3. Science Self-Efficacy and Academic Self-Concept

Research by Chemers and colleagues [16] found a strong relationship between science identity and science self-efficacy. Self-efficacy is an individual’s belief in their ability to complete a task or achieve a goal [26]. The current study focuses on science self-efficacy, a subcategory that focuses on an individual’s confidence in their abilities to complete science-related tasks such as using scientific language, developing a research question, and interpreting scientific data [16].

Research has found that UREs can promote students’ ability to think like scientists, understand the research process, and conduct scientific tasks [21–23]. Hunter et al. [13] found that students who participated in a URE grew their confidence in conducting research and contributing to science. Moreover, Lo and Le [27] found that STEM students’ participation in a course-based undergraduate research experience (CURE) increased research skill abilities such as collecting and analyzing scientific data, composing a research paper, and reading scientific literature [27]. Science self-efficacy is a significant predictor of students’ aspirations for research careers [28], commitment to a science career [16], and persistence in STEM disciplines [29]. Strong science self-efficacy also leads to a stronger science identity [29].

URE participation has also shown to improve a student’s academic self-concept [10]. Bong and Skaalvik [30] define academic self-concept as an individual’s belief in their perceived ability to perform specific academic and educational tasks. In the study by Betz and colleagues [10], URE participants, who were mostly students from URGs, increased scores on all academic self-concept items, which included items asking them to rate their confidence in their academic knowledge and abilities, their capability to achieve success in their education, and be successful in a career after graduation [10].

### 1.4. Sense of Belonging

Among college students, prior research identified sense of belonging as a significant predictor of academic success and motivation [31,32]. Sense of belonging in higher education refers to a student’s perception of social support on campus, feelings and perceptions of connectedness, or feelings of being accepted and valued as a campus community member [31] (p. 4). A sense of belonging has “cognitive and affective elements” [33] (p. 328), whereby students’ reflection (cognition) on their role in relation to the group shapes how they feel. Undergraduates who report a greater sense of belonging are more likely to report interest and persist in STEM majors or careers than students who leave STEM [5,34,35]. However, women and students from URGs in STEM majors are more likely to report a lower sense of belonging than men and White students [35–37].

### 1.5. COVID-19

UREs are traditionally delivered in person and on campus [38]. However, in March 2020, the COVID-19 pandemic forced many college campuses in the U.S. to close and temporarily shift all learning and training online [38]. This shutdown caused major disruptions to institutions of higher education, as in-person summer undergraduate research experience (SURE) programs, such as the National Science Foundation’s (NSF) Research Experiences for Undergraduates (REU), Summer Research Opportunity Programs (SROP), Summer Undergraduate Research Fellowships (SURF), and the National of Institutes of Health’s numerous programs, were forced to shift all training online or cancel their programs. For example, of the 125 NSF-funded REU programs, only 25 (20%) pivoted their summer training online, whereas 100 (80%) canceled their summer programs in response to COVID-19 [38]. These NSF-funded REU programs typically provide summer experiences to over 1200 students, of whom 70% are women and 60% are students from URGs. Given that these programs heavily recruit women and URG students, these students were disproportionately affected by summer program cancellations. As a result, these students lost access to months of paid hands-on research experience, networking opportunities, graduate school preparation, and professional development.

According to Erickson and colleagues [38], among the UREs that pivoted online, participants reported positive mentorship and increased knowledge and skills. They also reported feeling connected to their peers, the URE program, or the scientific community. However, half of the URE students surveyed felt isolated or disconnected, and more than half struggled with the lack of structure related to remote research. Furthermore, nearly 70% of students in the UREs reported inconsistent communication among participants, program leaders, and their mentors [38]. These findings suggest that differences in URE design may lead to different URE student experiences and outcomes.

For example, the Mayo Clinic’s summer URE provided a four-week intensive training program for 170 undergraduate students and intentionally supported students’ mental well-being [39]. Overall, these students reported increased confidence in their research skills and increased knowledge of and interest in biomedically-based research careers. Students also reported a decrease in stress, as well as gains in resilience and life satisfaction, suggesting that core objectives of traditional in-person UREs can be accomplished remotely and positively impact students’ well-being. Addressing student well-being was critical during the COVID-19 pandemic as many students experienced learning loss, isolation, increased stress, and anxiety during this period [40–42]. Given the ability of UREs to increase science-related outcomes, resilience, and life satisfaction, it is important to examine how URE programs impacted student outcomes over time during the COVID-19 pandemic.

### 1.6. BUILD PODER Program Overview

The current study will focus on student outcomes among participants in a large undergraduate biomedical research training program known as Building Infrastructure Leading to Diversity (BUILD) Promoting Opportunities for Diversity in Education and Research (PODER) program at California State University, Northridge (CSUN). Launched in 2014, the National Institute of General Medical Sciences’ BUILD Initiative funded ten institutions to engage, support, and retain undergraduate students from diverse backgrounds in biomedical/behavioral sciences and other STEM-related fields. BUILD recognizes 87 majors in biological and life sciences, engineering, health professions, and physical and social sciences [43]. The BUILD program is grounded in Critical Race Theory (CRT) to create an inclusive environment for students and faculty in mentored research experiences.

BUILD PODER’s goal is to increase undergraduate students’ science self-efficacy, confidence, science identity, and interest in pursuing biomedical/behavioral-related graduate programs. To achieve this goal, BUILD PODER affiliated faculty at CSUN are trained to be CRT-informed mentors and to improve their own sense of science identity and overall satisfaction in their research, all of which leads to more research productivity and improved scholarship.

To provide student training, BUILD PODER supports various activities throughout the academic year. The summer before their first year in BUILD, students participate in a four-week intensive entry-to-research training program where they receive skills training, professional development, learn critical race theory and cultural competence, participate in team-building activities, and enter a supportive community environment, which is maintained throughout the academic year. Students work closely with CRT-trained faculty mentors to conduct research in their related disciplines, attend conferences, and disseminate research findings. BUILD PODER students are matched with their faculty mentors using a rigorous pairing process to ensure that students are well-matched with mentors. During the student’s second summer in the URE, students are required to participate in research experiences with BUILD-affiliated research partner institutions (e.g., UCSD STARS, Stanford SSRP, USC Bridging the Gaps) or another summer URE (e.g., Summer Research Opportunity Programs (SROP) and the NSF’s Research Experience for Undergraduates (REU).

Students must attend biweekly meetings during the academic year with all students in the BUILD PODER program and the student training directors for support, workshops, and seminars to cover topics such as community-engaged research, social justice, and career paths. In addition, students participate in BUILD PODER-sponsored social events (e.g., game nights and movie nights). In addition, this program provides its students with degree progress advising, 60% tuition assistance, graduate school preparation, professional development training, and monthly stipends to all students.

### 1.7. Current Study

To understand COVID-19’s impact on undergraduate learning and research training, the current study examined how science identities, research skills, and perceptions of academic ability changed from 2019 to 2020 among different groups of undergraduates. In addition, this study will describe how the BUILD URE program pivoted online in response to the COVID-19 pandemic. The following research questions guided the study:

1. How did COVID-19 impact science identity, science self-efficacy, and academic self-concept scores from 2019 to 2020 among biomedical science majors?
2. Are there differences in science identity, science self-efficacy, and academic self-concept scores across time from 2019 to 2020 between students participating in BUILD and non-BUILD students?
3. Are there differences in science identity, science self-efficacy, and academic self-concept scores across time from 2019 to 2020 among URG and non-URG students?
4. In 2020, were there differences in sense of belonging scores between students participating in BUILD and non-BUILD students?

## 2. Methods

### 2.1. Participants and Procedure

Surveys were completed by students at CSUN, a large public university in Southern California. In Spring 2019, current undergraduates were invited to take the Student Annual Follow-Up Survey (SAFS) [44,45]. The SAFS was developed and administered by the Coordination and Evaluation Center (CEC) at the University of California, Los Angeles [46]. The SAFS included measures of science identity, science self-efficacy, and academic self-concept. The survey took around 25 min to complete. Participants received a $25 Amazon gift card for completing the study. All particpants provided informed consent.

Students who completed the 2019 SAFS and expected to graduate in 2020 were invited to complete the 2020 College Senior Survey (CSS). The CSS was administered online between 14 February 2020, and 30 June 2020. The CSS was developed and administered by the Cooperative Institutional Research Program (CIRP) at the Higher Education Research Institute (HERI) at the University of California, Los Angeles (UCLA) [47]. The CSS included measures of science identity, science self-efficacy, academic self-concept, and sense of belonging and took approximately 25 min to complete. Participants received a $25 Amazon gift card for completing the study. All participants provided informed consent before they participated in the study. All surveys and related protocols were approved by the Institutional Review Board (IRB) at UCLA, which has a reliance agreement with CSUN to administer the SAFS and the CSS.

### 2.2. Participant Characteristics

Participants who completed both the 2019 SAFS and the 2020 CSS served as the initial sample for this study. Among these participants, students who did not report a bioscience-related major in the CSS survey were removed from the analysis (See Supplemental File for list of bioscience-related majors). Students participating in BUILD PODER identified and coded as the intervention group. Propensity score matching (PSM) was then used to estimate propensity scores based on four matching estimators: (1) formal training or workshop experience, (2) conducting own scientific research, (3) honors and awards, and (4) scientific conference attendance or presentations). PSM is used to reduce selection bias [48], such as the fact that the intervention effects may not be caused by the program’s effectiveness but by unobserved differences between those who chose to participate in the intervention and those who chose not to. Based on the PSM analysis, 133 participants produced similar propensity scores. Of the 133 participants, 16% (*n* = 22) were BUILD students, and 83% (*n* = 111) were non-BUILD students. PSM analyses were conducted using SPSS 25.0 (IBM SPSS Statistics for Windows, 2017). In addition, the Office of Institutional Research (IR) at CSUN provided supplemental student demographic information, which included self-identified binary gender and whether the students self-identified as belonging to a race/ethnicity that is traditionally underserved racial/ethnic group (URG: e.g., Hispanic, Black/African American, Pacific Islander, Native Hawaiian, American Indian, Alaskan Native). Across students in our sample, 53.4% of students (*n* = 71) self-identified as female, and 42.9% of students (*n* = 57) self-identified as being from a traditionally underserved racial/ethnic group (URG).

### 2.3. Outcome Variables

Science identity was assessed using a 4-item scale [49]. Students were asked to what extent the following statements were true, for example, “I have come to think of myself as a scientist” and “I feel like I belong to the field of science.” Responses were coded 1 = “strongly disagree”, 2 = “disagree somewhat”, 3 = “neutral”, 4 = “agree somewhat”, and 5 = “strongly agree” (See Supplementary Files for all survey items).

Science self-efficacy was assessed using a 6-item scale [49]. Students rated how confident they felt about completing various activities, including “generating a research question”, “determining how to collect appropriate data”, and “explaining the results of a study.” Responses were coded 1 = “not at all”, 2 = “somewhat”, 3 = “moderately”, 4 = “very”, and 5 = “absolutely” (See Supplementary Files for all survey items).

Academic Self-Concept was assessed using a 4-item scale from a 20-item scale measuring self-ratings related to academic and personality traits [49]. Students rated themselves on the following traits compared to the average person their age. Students rated their “academic ability”, “drive to achieve”, mathematical ability”, and “self-confidence (intellectual).” Responses were coded 1 = “lowest 10%”, 2 = “below average”, 3 = “average”, 4 = “above average”, and 5 = “highest 10%” (see Supplementary Files for all survey items).

Sense of Belonging was assessed using a 4-item scale, which was taken from a 16-item scale measuring institutional opinion [49]. Students were asked to indicate the extent to which they agree or disagree with the following statements, which included “I feel a sense of belonging to this campus”, “If asked, I would recommend this college to others”, “I will give this college money as an alum”, and “I feel I am a member of this college”, Responses were coded 1 = “strongly disagree”, 2 = “disagree”, 3 = “agree”, and 4 = “strongly agree”.

### 2.4. Reliability and Validity

The SAFS and the CSS measured science identity, science self-efficacy, and academic self-concept using identical survey items, allowing us to analyze change over time. Sense of belonging data from the CSS were included in the analysis. Reliability and validity were checked for all measures. For reliability, Cronbach’s alpha (α), separation index, and reliability index from item response theory (IRT) were examined using ConQuest 5.12.3 and Winsteps 5.4.2. According to Boone et al. [50], a Cronbach’s α more than 0.7, a separation index of more than 2.0, or a reliability index of more than 0.8 was considered to have strong internal consistency, high separation, or high reliability (See Supplementary Files for reliability values). A confirmatory factor analysis (CFA) was conducted using Mplus 8.9 for validity. Factor loadings were above the minimum accepted threshold of 0.30 and suggest items loaded onto the same latent constructs [51], which means that all these 14 items were adequate to measure the latent constructs used for this analysis (i.e., science identity, science self-efficacy, and academic self-concept) (See Supplementary Files for factor loadings).

### 2.5. Analysis Plan

We used repeated measures between within-subjects multivariate analysis to examine the change in science identity, science self-efficacy, and academic self-concept from 2019 to 2020. We added BUILD participation as a between-subjects factor to examine any significant differences between BUILD participation and non-BUILD participation regarding the three outcome variables. In addition, mean sense of belonging scores between BUILD and non-BUILD participants was compared using *t*-tests, and we examined the correlations between sense of belonging and the three outcome variables. The analyses were conducted in SPSS (version 25.0).

### 2.6. Redesigning the BUILD PODER for the Online Environment

Describing how BUILD PODER shifted to the online environment in response to COVID-19 is important, as it demonstrates that UREs can be redesigned and pivot online if there are disruptions to in-person learning and training. In addition, challenges and lessons learned from the redesign process are described. Due to local stay-at-home ordinances on 19 March 2020, CSUN shifted classes online to prevent the spread of COVID-19. In one week, BUILD PODER administrators and staff worked together to shift all components of the BUILD program (i.e., weekly meetings, mentoring, and training) online. Additionally, BUILD had to rethink and adapt its four-week summer entry-to-research training program for the incoming 2020 student cohort.

#### 2.6.1. Student Support and Community Building

Being equity-minded was crucial to ensure remote students did not miss on the same training experiences of previous cohorts. During the academic year, all BUILD meetings, including the bi-weekly meetings, were converted to synchronous online meetings using Zoom. Despite moving online, BUILD continued hosting guest speakers and student alums panels. In addition, BUILD continued to host online community-building group activities (e.g., paint nights, game nights, and movie nights) and health-related sessions (e.g., yoga, meditation, mindfulness) on Zoom.

#### 2.6.2. Faculty Mentorship

Faculty mentors were asked to provide research training online, a challenging task for several research labs. Therefore, BUILD created a list of 40 online student training activities for faculty members, which BUILD provided at no cost. These supplemental training opportunities included training in Python, Excel, R studio, statistics, writing and dissemination, PowerPoint, and public speaking.

#### 2.6.3. Summer Research Training

Continuing BUILD students are expected to participate in research over the summer and present their findings at professional conferences. Typically, students participate in research projects outside CSUN with many traveling nationwide. Due to COVID-19, in 2020 only 7 of the 42 continuing students had summer URE programs that pivoted to online; the rest of the summer UREs were canceled. As a result, our BUILD partnerships became vital to student summer training in 2020. We relied on our Stanford and UC San Diego research partners to provide online summer UREs. Two research partners, Stanford’s Summer Research Program (SSRP) and UCSD’s Summer Training Academy for Research Success (STARS), converted their programs to remote training. The BUILD Research Collaboratory (BRC)—a summer data research program organized by the San Francisco State BUILD program, also provided additional training for some students. At CSUN we collaborated with our BUILD-affiliated faculty to create COVID-related summer URE projects. We asked several faculty mentors to extend their academic training through the summer. California State University, Northridge faculty in health sciences and psychology departments developed COVID-19-related research projects. Ultimately, through our partnerships and BUILD faculty, all 42 continuing students had online summer research opportunities for the summer of 2020.

All incoming BUILD PODER students are required to complete a four-week entry-to-research training program, known as Summer JumpStart. For the incoming 2020 cohort, Summer JumpStartconsisteds of synchronous and asynchronous training workshops focusing on research skills development, research ethics training, mentoring relationships, professional conduct, and maintaining well-being. To prevent “Zoom fatigue”, we divided each day’s programming into three-to four-hour modules, followed by “On-Your-Own” modules consisting of prerecorded videos and assignments students completed independently. All BUILD PODER principal investigators and staff worked to facilitate student workshops. BUILD PODER participants were also introduced to bioinformatics and were trained to create research posters and prepare and present oral presentations.

In addition, for all BUILD PODER participants, program staff prepared “goodie bags”, which contained everything needed for the entry-to-research program. For example, one bag contained tools required for research and methods training. For the health sciences and social science students, their bag included manuals for software such as SPSS and NVivo, and an American Psychological Association (APA) manual. These tools assisted students with their summer research training and introductory courses in social science research, including qualitative and quantitative methods. For biological science students, bags contained lab supplies, including a microscope and reagents to perform DNA extractions, supplies to make agar LB plates to culture bacteria, swabs for microbes, isolate microbes, and supplies to perform Gram staining to identify and differentiate between monoderm and diderm bacteria.

To develop a sense of community and belonging, all students received a second bag with individually wrapped marked packets with BUILD PODER “swag”, such as branded face masks, lanyards, water bottles, and a t-shirt. This bag also contained popcorn and candy for movie nights and a painting kit for a virtual paint night. Staff created intentional, engaging, and interactive community-building activities. Social activities ranged from competitive games such as the BUILD version of Family Feud to critical race theory-focused activities such as the privilege spectrum exercise. Summer JumpStart also hosted weekly movie nights on Zoom, and a virtual Paint Night, led by a local artist, to paint a young scientist of color. Hosting the annual summer field trip was the most difficult activity to adapt online. The annual Summer JumpStart field trip was developed by CSUN faculty members in geography, health sciences, and history. It provided students with an interdisciplinary Virtual Toxic Tour of the surrounding community.

## 3. Results

### 3.1. Biomedical Students

When pooling all three outcomes, there was an overall significant change in student science outcomes across years (*F*_(3, 127)_ = 5.55, *p* < 0.01). There was a significant change in science self-efficacy (*M*_19_ = 19.89, *SE* = 0.56; *M*_20_ = 21.87, *SE* = 0.65, *p* < 0.001) and a significant change in academic self-concept (*M*_19_ = 14.63, *SE* = 0.26; *M*_20_ = 15.14, *SE* = 0.27; *p* = 0.03). However, there was not a significant change in science identity (*M*_19_ = 14.33, *SE* = 0.419; *M*_20_ = 14.85, *SE* = 0.49; *p* = 0.09). Means and mean differences for all measures are displayed in Table 1.

### 3.1. BUILD Participation

When comparing BUILD and non-BUILD students, the tests of within-subjects effects showed a significant interaction between the change in overall student outcomes and BUILD participation (*F*_(3, 127)_ = 2.72, *p* < 0.05). Between BUILD and non-BUILD students, there was a significant change in science self-efficacy (*F*_(3, 129)_ = 5.767, *p* < 0.05) (Table 2). However, between BUILD and non-BUILD students, there was not a significant change in science identity (*F*_(3, 129)_ = 3.01, *p* = 0.09) or academic self-concept (*F*_(3, 129)_ = 1.67, *p* = 0.20). Accompanying Figures S1–S3 can be found in the Supplementary Files.

### 3.2. URG Status

The tests of within-subjects effects did not show a significant interaction between the change in overall student outcomes and URG status (*F*^(3, 127)^ = 1.434, *p* = 0.236). When comparing URG and non-URG students, there was not a significant change in science identity (*F*^(1, 129)^ = 1.708, *p* = 0.194), science self-efficacy (*F*^(1, 129)^ = 2.734, *p* = 0.101), or academic self-concept (*F*^(1, 129)^ = 1.102, *p* = 0.296) (Table 3). The results of all repeated measures within-subjects analysis are displayed in Table 3.

### 3.3. Sense of Belonging

BUILD students (*Mean* = 12.06, *SD* = 2.29) reported a higher sense of belonging than non-BUILD students (*Mean* = 11.39, *SD* = 2.71); however, the difference was not statistically significant (*t*_176_ = −1.35, *p* = 0.18).

For BUILD students, there were significant correlations between sense of belonging and science identity, *r* = 0.43, *p* < 0.01, between sense of belonging and science self-efficacy, *r* = 0.35, *p* < 0.05, and a moderate but non-significant correlation between sense of belonging and academic self-concept, *r* = 0.33, *p* = 0.06. In contrast, for non-BUILD students, there were no significant correlations between sense of belonging and science identity, *r* = 0.15, *p* = 0.07, or between sense of belonging and science self-efficacy, *r* = 0.05, *p* = 0.58. However, there was a significant correlation between sense of belonging and academic self-concept, *r* = 0.26, *p* < 0.01. The correlation matrix is displayed in Table S4 in Supplementary Files.

## 4. Discussion

The current study examined how COVID-19 impacted science identity, science self-efficacy, and academic self-concept scores from 2019 to 2020 among biomedical science majors while also comparing changes over time between (1) BUILD students and non-BUILD students and (2) URG students and non-URG students. In addition, sense of belonging scores from 2020 were compared between BUILD and non-BUILD students. Results indicate that the students in BUILD significantly increased science self-efficacy scores, unlike non-BUILD students. In addition, changes over time between URG and non-URG students were not significant, and differences in sense of belonging scores between BUILD and non-BUILD students were not significant.

While past studies compared pre-pandemic to pandemic URE outcomes [52], this was the first study to use longitudinal data to compare outcomes between URE students and non-URE students and between URG and non-URG students from 2019 to 2020. Examining undergraduate student outcomes during the COVID-19 pandemic is critical, as research indicates that college students, compared to the general population, were at greater risk for poorer mental health, poorer psychological well-being, and higher levels of emotional loneliness during 2020 [41]. These negative health outcomes may be related to the abrupt shift to online learning, as research found that some of the most common concerns among a sample of college students whose courses were shifted fully online in March 2020 were that they would not be able to complete the academic year, would not be able to graduate on time, and were worried about their health and the health of family members [40]). In addition, some students reported lower scores of university or college staff engaging students online and lower scores of interacting successfully with lectures during online teaching [40]. Though remote learning allowed students to socially distance and reduce their exposure to COVID-19, it also impacted mental health and academic concerns among many students, and it is important to note that many impacts of COVID-19 differed by race and ethnicity [53].

For example, COVID-19 infection and hospitalization rates in the U.S. varied by race and ethnicity, such that COVID-19 infection and Intensive Care Unit (ICU) admissions were higher among African American, Hispanic/Latinx, and Asian Americans compared to Whites [54]. These disparities result from longstanding inequities related to lower income, risker job conditions, language barriers, being uninsured, having higher rates of chronic health conditions, and receiving low-quality health care [53]. The URE program described in the current study is housed at a Hispanic-serving institution (HSI), where 56.07% of students are Hispanic/Latinx [55]. Examining undergraduate student outcomes during the COVID-19 pandemic is important, particularly among priority populations at higher risk for adverse health and academic outcomes related to COVID-19.

### 4.1. Science Self-Efficacy

Science self-efficacy is a significant predictor of students’ aspirations to pursue a research-related career [28] and an important predictor of their commitment to science careers [16]. Our results indicate that despite a growing global pandemic, BUILD PODER students significantly improved a critical student outcome from 2019 to 2020. These findings are consistent with past research that found UREs can increase students’ abilities to think scientifically, understand the research process, and conduct research projects [21,22,56].

For the science self-efficacy measure, students rated themselves on specific science and research-related tasks and skills, such as generating a research question, data collection, and explaining study results. Across all biomedical students, the most significant change was in science self-efficacy, compared to science identity or academic self-concept. The early months of the COVID-19 pandemic were a period of major adjustment, isolation, and uncertainty. Therefore, students may have found it difficult to accurately assess their membership in the scientific community or how they compared to their peers if they were socially isolated. Additionally, a significant change in science self-efficacy among students, but not science identity, may be explained by science outcomes not always developing concurrently [57]. Carlone and Johnson [19] describe science identity as consisting of three components, two of which focus on using scientific skills and understanding scientific concepts, similar to science self-efficacy. A student’s confidence in scientific and research skills may develop sooner than science identity. Developing one’s identity as a scientist is informed by internal and external factors, including recognition by the self and others as a scientist, which may take longer to recognize, assess, and accept.

The context in which BUILD PODER students significantly increased science self-efficacy scores compared to non-BUILD students is important. From 2019 to 2020, BUILD students had an existing support system of peers, faculty, and staff to help them navigate academia, build community, and gain research training. In response to the COVID-19 pandemic, BUILD shifted all training and activities online, providing its students with continued research training and support. Despite the campus closures, during the early months of the pandemic, students continued their research online with their BUILD mentors, met biweekly with all BUILD students online, and attended seminars remotely. For continuing BUILD students whose summer URE programs were canceled, we offered a summer online training opportunity with BUILD-affiliated research partners (i.e., UCSD STARS, Stanford SSRP) or BUILD faculty members. For new students joining BUILD PODER in the summer of 2020, the training program pivoted to online, with the same training objectives as the traditional in-person programs to provide the new BUILD cohort with similar research training opportunities as prior cohorts. Survey data did not indicate whether non-BUILD students participated in research activities. However, the slight increase in science self-efficacy from 2019 to 2020 among non-BUILD students may be due to biomedical-related coursework or membership in a research lab.

### 4.2. Science Identity and Academic Self Concept

Though BUILD PODER students reported higher levels of science identity and academic self-concepts in 2019 and 2020 compared to non-BUILD students, we did not see significant increases in scores for either group. These findings are similar to Supriya et al.’s [58] study on undergraduate biology students during COVID-19, which found that students reported various negative impacts related to understanding course content, peer and faculty interactions, a sense of the biology community on campus, and career preparation. In addition, women were more likely to report negative impacts on learning and career preparation, and Pell-grant-eligible students (e.g., lower-income students) reported decreases in peer interactions.

In 2019, science identity scores for BUILD students were higher (*M* = 16.81) than for non-BUILD students (*M* = 11.89). In addition, for BUILD students, science identity scores improved from 2019 to 2020, unlike non-BUILD students, who saw a slight decline in science identity. Though these improvements were not significant, these findings are important, as previous research found that STEM majors who experience declines in science identity over their college career are less likely to pursue a STEM-related career [25]. These findings indicate the importance of URE participation among biomedical science students. Furthermore, these findings suggest that BUILD participation may protect students against declining levels of science identity, which may have been related to the uncertainty and academic disruptions during the early months of the COVID-19 pandemic.

Previous studies identified factors that can help cultivate science identity, such as being recognized and validated by faculty, mentors, and peers [19]. However, these interactions were disrupted and altered by the abrupt shift to online learning and training, where it may have been more difficult to build and maintain meaningful relationships with faculty and peers, including those in biomedical research. BUILD students’ science identities were already higher than non-BUILD students. It may be that non-BUILD students were not as active in research experiences, which are known to be high-impact practices [59] that impact students’ overall undergraduate experiences.

However, shifting to remote learning and training may have impacted all students’ feelings about academic abilities and career preparation. Given that the survey was collected during the early part of the COVID-19 pandemic, when many universities were closing campuses, suspending in-person classes, suspending in-person research, implementing hiring freezes, or canceling summer research programs, students in the current study may have been worried about completing biomedical courses online, graduating on time or pushing back their graduation date, which may have impacted plans for internships, graduate school, or job searches.

Regarding academic self-concept, neither BUILD nor non-BUILD students differed over time. Academic self-concept asked students to rate themselves on their academic ability, mathematical ability, drive to achieve, and intellectual self-confidence. It is possible that despite efforts to engage BUILD students throughout the summer and academic year through their biomedical-related coursework, students were not successfully engaged [40]. Additionally, students may not have been able to interact successfully with faculty and staff during online teaching, impacting perceptions of academic self-concept and sense of belonging on campus.

### 4.3. Sense of Belonging

While sense of belonging did not differ between BUILD and non-BUILD students, it must be noted that there were stark differences in the relationship between sense of belonging and science outcomes for BUILD students compared to non-BUILD students. Sense of belonging was significantly related to BUILD students’ science identities and self-efficacy. In contrast, for non-BUILD students, sense of belonging was significantly related to their academic self-concept. Therefore, it is possible that for students in this URE, sense of belonging indirectly affected their scientific outcomes, and programmatic activities planned by the BUILD program indirectly impacted the overall experiences of the URE student.

### 4.4. Limitations

This study was limited to undergraduates in Southern California. Therefore, results may not be generalizable to undergraduates in other regions of the United States. In addition, the BUILD URE was designed for students majoring in biomedical science, which limits the generalizability of this program to other academic disciplines. Students in the analyses were identified as BUILD participants or non-BUILD participants. However, survey data did not indicate whether non-BUILD students participated in other URE programs on campus. Non-URE students in our study may have been engaged in research activities, had a faculty mentor, or even had a faculty mentor with BUILD training. Therefore, future research should examine the research activities of non-URE students to determine if they participated in mentored research activities or course-based research, if at all.

Our study collected data from February 2020 to June 2020, during the early months of the COVID-19 pandemic. Therefore, our results may not be generalizable to other periods of the pandemic. The 2020 CSS dataset did not include information about the date and time surveys were completed. Our study was not able to examine sense of belonging over time. Unfortunately, the 2019 SAFS only required first-year students to complete measures on sense of belonging. Given that a sense of belonging is an important predictor of academic success and motivation among college students [31,32], future research should compare sense of belonging between URE students and non-URE students before and during the COVID-19 pandemic to examine how feelings of belonging to their college campus were impacted.

With the current findings, we suggest that science outcomes may not be developing concurrently [57]. Future research should examine students over a longer period of time to determine if students’ science identities and academic self-concepts continue to grow over time beyond their time in science-related UREs, such as in graduate studies and the workforce.

## 4.5. Conclusions

Given the importance of science self-efficacy and science identity in an undergraduate student’s intention to actively pursue a career in biomedical science, our results indicate that URE programs such as BUILD were able to improve and/or maintain critical student outcomes during a global pandemic, despite nationwide shifts to online education, which compromised student engagement. The early months of the COVID-19 pandemic were a period of major adjustment, isolation, and uncertainty for all students, which may explain why there were few to no significant increases in science identity and academic self-concept from 2019 to 2020 when students were in their senior year and about to graduate. These results highlight the importance of URE participation among college students in biomedical science majors, particularly during the pandemic.

## Supplementary Material

Supplemental Files

## Figures and Tables

**Table 1.** Pairwise comparisons of science identity, science self-efficacy, and academic self-concept.

Construct	Mean (Time 1)	Mean (Time 2)	Mean Difference (T2 − T1)	Std.Error	*p* Value
Science Identity	14.33	14.85	0.52	0.31	0.09
Science Self-Efficacy	19.89	21.87	1.98 ***	0.55	0.00
Academic Self-Concept	14.63	15.14	0.51 *	0.23	0.03

*Note. p* values are Bonferroni adjusted for multiple comparisons.

* *p* < 0.05.

*** *p* < 0.001.

**Table 2.** Pairwise comparisons of science identity, science self-efficacy, and academic self-concept stratified by BUILD participation.

Construct		Mean (Time 1)	Mean (Time 2)	Mean Difference (T2 − T1)	*p* Value
Science Identity	non-BUILD BUILD	11.89 16.81	11.83 17.87	−0.02 1.06	0.08
Science Self-Efficacy	non-BUILD BUILD	17.81 21.98	18.48 25.26	0.67 3.28 *	0.01
Academic Self-Concept	non-BUILD BUILD	14.15 15.11	14.37 15.92	0.22 0.81	0.19

Note.

* *p* < 0.05.

**Table 3.** Summary of repeated measures within-subjects analysis.

	Source		Sum of Squares	*Df*	Mean Square	*F*	*p*	Partial Eta Squared
time	SI SSE ASC	Time 1 vs. Time 2 Time 1 vs. Time 2 Time 1 vs. Time 2	9.88 141.61 9.55	1 1 1	9.88 141.61 9.55	2.81 13.17 *** 5.02 *	0.09 0.00 0.02	0.02 0.09 0.03
time ∗ intervention	SI SSE ASC	Time 1 vs. Time 2 Time 1 vs. Time 2 Time 1 vs. Time 2	10.56 61.99 3.17	1 1 1	10.56 61.99 3.17	3.01 5.76 * 1.67	0.08 0.01 0.19	0.02 0.04 0.01
time ∗ URG	SI SSE ASC	Time 1 vs. Time 2 Time 1 vs. Time 2 Time 1 vs. Time 2	5.99 29.39 2.09	1 1 1	5.99 29.39 2.09	1.70 2.73 1.10	0.19 0.10 0.29	0.01 0.02 0.00
Error (time)	SI SSE ASC	Time 1 vs. Time 2 Time 1 vs. Time 2 Time 1 vs. Time 2	452.46 1386.62 245.11	129 129 129	3.50 10.74 1.90			

Note.

*** *p* < 0.001.

* *p* < 0.05.

SI = Science Identity, SSE = Science Self-Efficacy, and ASC = Academic Self-Concept. Intervention = BUILD participation. URG represents a traditionally underserved racial/ethnic group.

## Data Availability

Data available on request due to restrictions: The data presented in this study are available on request from the corresponding author. The data are not publicly available due to privacy restrictions.
