# Supplementary material for: The Impact of the COVID-19 Pandemic on Sense of Belonging and Science Outcomes among Biomedical Science Students: A Longitudinal Study
Source: Educ Sci (Basel). Author manuscript; Available in PMC 2024 Oct 17. (PMC11484766; doi:10.3390/educsci13060579)
Supplement: Supplemental Files [file NIHMS2023165-supplement-Supplemental_Files.pdf]

**Supplementary Materials**

**Table S1: Biomedical Science majors at California State University, Northridge (CSUN) that qualify for BUILD PODER participation**

| CSUN Colleges | BUILD Eligible Majors |
|---|---|
| Engineering & Computer Science | |
| | Computer Science |
| | Electrical & Computer Engineering |
| | Mechanical Engineering |
| Health & Human Development | |
| | Child and Adolescent Development |
| | Communication Disorders and Sciences: Speech & Hearing Sciences |
| | Environmental and Occupational Health |
| | Family and Consumer Sciences: Dietetics & Food |
| | Health Sciences |
| | Kinesiology |
| | Nursing |
| | Physical Therapy |
| Science & Mathematics | |
| | Biology |
| | Chemistry & Biochemistry |
| | Geological Sciences |
| | Physics & Astronomy |
| | Mathematics |
| Social & Behavioral Sciences | |
| | Anthropology |
| | Geography and Environmental Studies |
| | Psychology |
| | Sociology |

**Study Measures**

**Table S2: Survey Items, Construct, Scale, and Reliability Indices**

| Item | Construct | Scale | SAFS19 Reliability Cronbach's $\alpha$/ Separation/ Reliability | CSS20 Reliability Cronbach's $\alpha$/ Separation/ Reliability |
|---|---|---|---|---|
| 1. I have strong sense of belonging to the community of scientists | **Science Identity** | 5-point scale | 0.87/ | 0.92/ |
| 2. I derive great personal satisfaction from working on a team that is doing important research | | | 2.57/ | 2.68/ |
| 3. I have come to think of myself as a scientist | | | 0.87 | 0.88 |
| 4. I feel like I belong in the field of science | | | | |
| 1. Use technical science skills (use of tools, instruments, and/or techniques) | **Science Self Efficacy** | 5-point scale | 0.93/ | 0.96/ |
| 2. Generate a research question | | | 3.02/ | 3.48/ |
| 3. Determine how to collect appropriate data | | | 0.9 | 0.92 |
| 4. Explain the results of a study | | | | |
| 5. Use scientific literature to guide research | | | | |
| 6. Integrate results from multiple studies | | | | |
| 1. Academic ability | **Academic Self-concept** | 5-point Likert scale | 0.75/ | 0.53/ |
| 2. Drive to achieve | | | 1.84/ | 1.38/ |
| 3. Mathematical ability | | | 0.77 | 0.66 |
| 4. Self-confidence (intellectual) | | | | |

**Table S3: Factor Loadings for Science Identity, Science Self-Efficacy, and Academic Self-Concept**

| | SAFS19 | | | CSS20 | | |
|---|---|---|---|---|---|---|
| Item | Science identity | Science self-efficacy | Academic self-concept | Science identity | Science self-efficacy | Academic self-concept |
| 1 | 0.79 | 0.64 | 0.80 | 0.86 | 0.78 | 0.69 |
| 2 | 0.51 | 0.79 | 0.65 | 0.76 | 0.88 | 0.68 |
| 3 | 0.86 | 0.84 | 0.56 | 0.93 | 0.94 | 0.33 |
| 4 | 0.80 | 0.87 | 0.62 | 0.88 | 0.89 | 0.63 |
| 5 | | 0.86 | | | 0.89 | |
| 6 | | 0.85 | | | 0.85 | |

*Note.* Factor loadings are all > .30

**Figure S1: Science Identity Trajectories across time between BUILD and non-BUILD students**

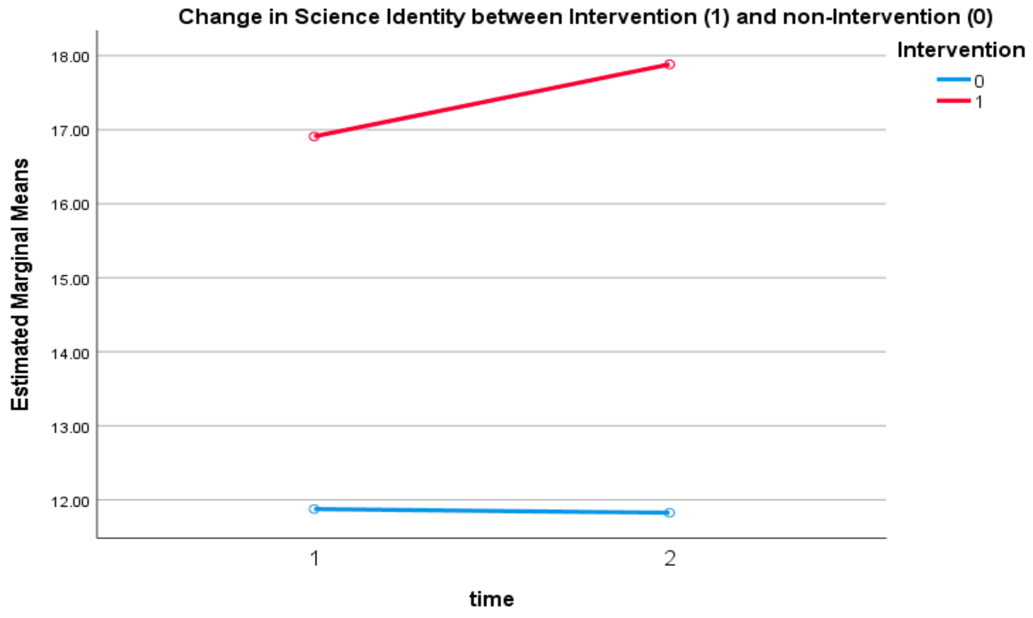

Figure S2: Science Self-efficacy Trajectories across time between BUILD and non-BUILD students

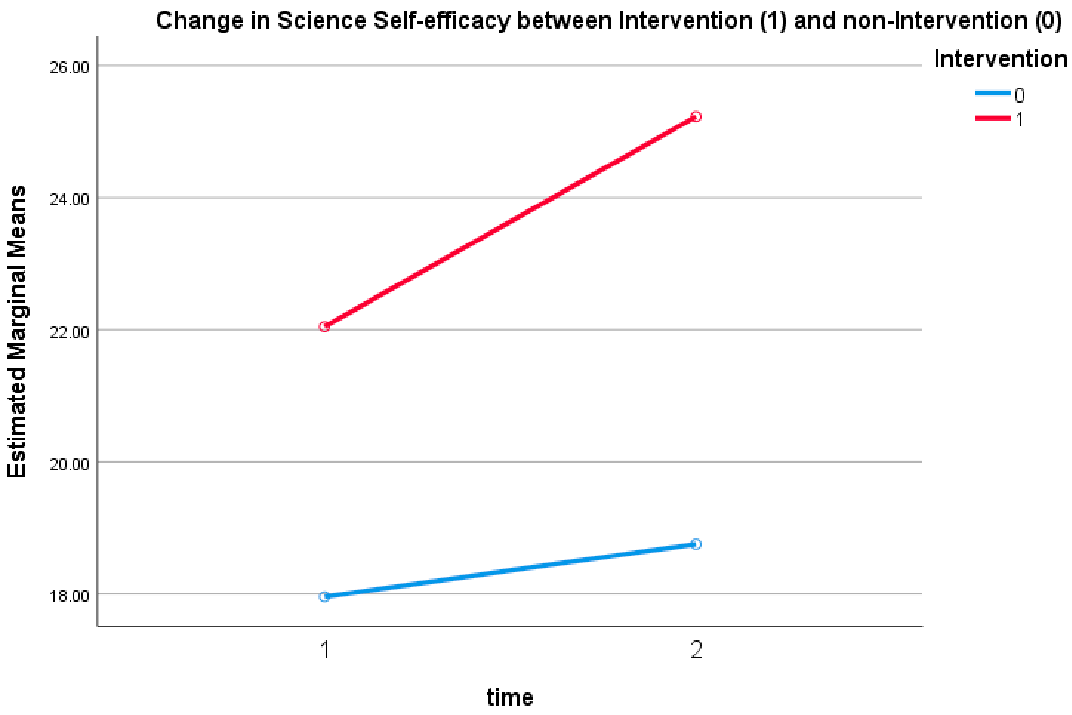


Figure S3: Academic Self-concept Trajectories across time between BUILD and non-BUILD students

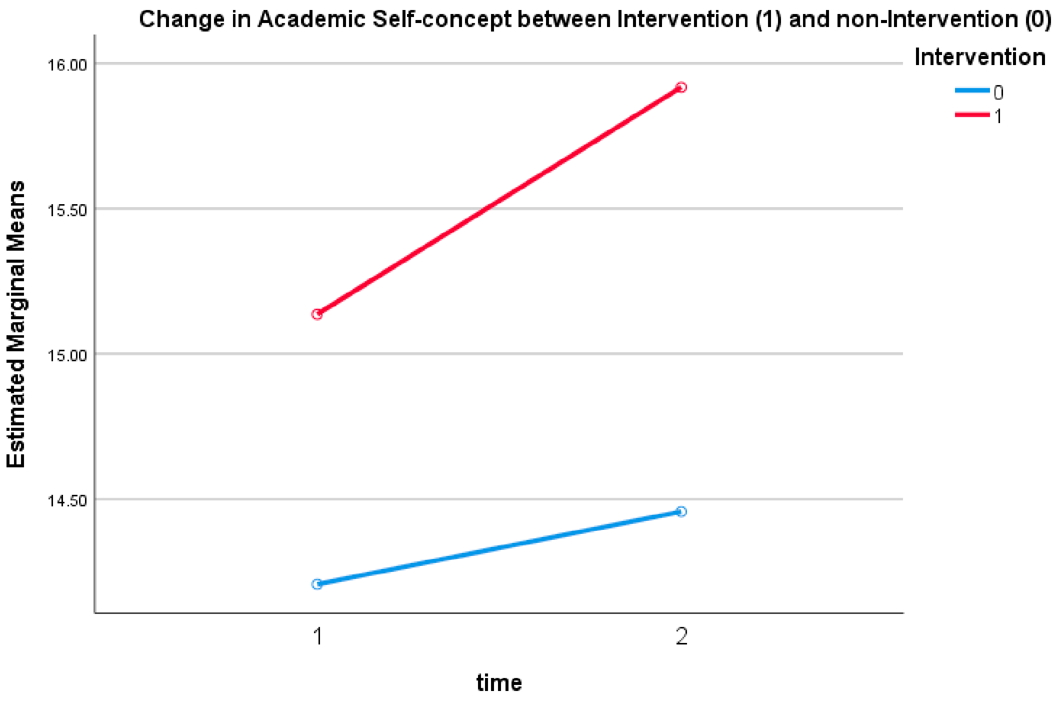

**Table S4: Correlation Matrix for Sense of Belonging**

| Item | BUILD | | | | Non-BUILD | | | |
|---|---|---|---|---|---|---|---|---|
| | **1** | **2** | **3** | **4** | **1** | **2** | **3** | **4** |
| **1. Sense of belonging** | - | | | | - | | | |
| **2. Science Identity** | **0.43**** | - | | | 0.15 | - | | |
| **3. Science Self-efficacy** | **0.35*** | 0.34* | - | | 0.48 | 0.62*** | - | |
| **4. Academic Self-concept** | 0.33 | 0.23 | 0.47** | - | **0.26**** | 0.30** | 0.35*** | - |
| *M* | 9.68 | 17.40 | 23.64 | 15.53 | 9.04 | 11.85 | 18.35 | 14.33 |
| *SE* | 0.44 | 0.77 | 1.00 | 0.45 | 0.21 | 0.34 | 0.45 | 0.20 |

*Note.* ***$p < .001$. **$p < .01$. *$p < .05$.
